# Detection and Validation of QTL Affecting Bacterial Cold Water Disease Resistance in Rainbow Trout Using Restriction-Site Associated DNA Sequencing

**DOI:** 10.1371/journal.pone.0138435

**Published:** 2015-09-16

**Authors:** Yniv Palti, Roger L. Vallejo, Guangtu Gao, Sixin Liu, Alvaro G. Hernandez, Caird E. Rexroad, Gregory D. Wiens

**Affiliations:** 1 National Center for Cool and Cold Water Aquaculture, ARS-USDA, 11861 Leetown Rd., Kearneysville, West Virginia, 25430, United States of America; 2 University of Illinois at Urbana-Champaign, Roy J Carver Biotechnology Center, 1201 W. Gregory Dr., Urbana, Illinois, 61801, United States of America; The Ohio State University, UNITED STATES

## Abstract

Bacterial cold water disease (BCWD) causes significant economic loss in salmonid aquaculture. Using microsatellite markers in a genome scan, we previously detected significant and suggestive QTL affecting phenotypic variation in survival following challenge with *Flavobacterium psychrophilum*, the causative agent of BCWD in rainbow trout. In this study, we performed selective genotyping of SNPs from restriction-site associated DNA (RAD) sequence data from two pedigreed families (2009070 and 2009196) to validate the major QTL from the previous work and to detect new QTL. The use of RAD SNPs in the genome scans increased the number of mapped markers from ~300 to ~5,000 per family. The significant QTL detected in the microsatellites scan on chromosome Omy8 in family 2009070 was validated explaining up to 58% of the phenotypic variance in that family, and in addition, a second QTL was also detected on Omy8. Two novel QTL on Omy11 and 14 were also detected, and the previously suggestive QTL on Omy1, 7 and 25 were also validated in family 2009070. In family 2009196, the microsatellite significant QTL on Omy6 and 12 were validated and a new QTL on Omy8 was detected, but none of the previously detected suggestive QTL were validated. The two Omy8 QTL from family 2009070 and the Omy12 QTL from family 2009196 were found to be co-localized with handling and confinement stress response QTL that our group has previously identified in a separate pedigreed family. With the currently available data we cannot determine if the co-localized QTL are the result of genes with pleiotropic effects or a mere physical proximity on the same chromosome segment. The genetic markers linked to BCWD resistance QTL were used to query the scaffolds of the rainbow trout reference genome assembly and the QTL-positive scaffold sequences were found to include 100 positional candidate genes. Several of the candidate genes located on or near the two Omy8 QTL detected in family 2009070 suggest potential linkages between stress response and the regulation of immune response in rainbow trout.

## Introduction

An important disease impacting salmonid aquaculture is bacterial cold-water disease (BCWD) [1,2]. The etiological agent of BCWD is a gram-negative bacterium, *Flavobacterium psychrophilum* (*Fp*), which also causes rainbow trout fry syndrome in small fish. Economic losses from *Fp* are due to direct mortality and also to deformities in fish that survive infection [2]. At present, the genetic and mechanistic basis of BCWD resistance in rainbow trout (*Oncorhynchus mykiss*) are not known. Initial efforts using phenotypic records and modeling, predicted 6–10 QTL in the NCCCWA odd-year spawning line [3]. In a subsequent QTL genome scan using ~300 microsatellite markers with an average genome marker density of ~6 cM in six families of either backcross or F_2_ intercross, we identified nine major QTL on seven chromosomes having moderate to large effects [4]. Interestingly, three of the QTL we detected for BCWD resistance based on the microsatellites genome scan appear to be co-localized with major QTL for handling stress response on chromosomes Omy8 and Omy12 that we have recently detected in the NCCCWA even-year spawning line [5,6]. In addition, we have recently employed RNA sequencing to identify genes differentially expressed between the ARS-Fp-R (resistant), ARS-Fp-C (control) and ARS-Fp-S (susceptible) rainbow trout lines that were derived from the same initial base population at the NCCCWA, as well as immune-related genes found to be expressed in all the genetic lines [7].

Single nucleotide polymorphism (SNP) markers are highly abundant bi-allelic loci which are evenly distributed across the genome. Several sequencing by genotyping procedures capable of simultaneous marker discovery and genotyping in many individuals have been recently developed for genome and genetic analyses [8]. One of them is restriction-site-associated DNA sequencing (RAD-seq) [9,10]. RAD-seq is an attractive method of SNP genotyping for species that lack genome resources because it does not require *a priori* marker discovery or a genome reference sequence. In recent years RAD-seq has been widely used in salmonid species for SNP discovery and for a variety of genome and genetic analyses [5,11,12,13,14,15,16,17,18,19,20,21].

In this study, we used selective genotyping of SNPs from *SbfI* RAD sequence data to validate the major QTL detected in two families from our previous work, increase the markers density in the QTL regions and possibly detect new QTL due to the higher marker density of the genome scan. The recently published rainbow trout reference genome [22] provides opportunities to integrate QTL mapping, genome sequence information, and gene expression data to identify candidate genes for BCWD resistance. Here, we used the genetic markers we found associated with or linked to BCWD resistance to query the scaffolds of the reference genome assembly and examined these regions for the presence of putative positional candidate genes based on differentially expressed genes and expressed immune-related genes identified in our previous RNA-seq study [7].

## Materials and Methods

### Ethics Statement

All animal and fish work has been conducted in accordance with national and international guidelines. The protocol for this study was specifically approved by the Institutional Animal Care and Use Committee (IACUC) of the US Department of Agriculture, Agricultural Research Service, the National Center for Cool and Cold Water Aquaculture (Protocol number 049). All efforts were made to ensure animal welfare and to minimize suffering.

### Rainbow trout rearing and challenge with *Fp*

Fish pedigree, rearing conditions and *Fp* challenges have been previously described [3]. Mortalities were removed and recorded daily. The fish health during the survival trial was monitored daily and no unexpected deaths were observed. Death of the fish collected daily was confirmed prior to fin clipping. Periodic sampling of the dead fish was conducted to make bacterial cultures and confirm the presence of *Fp* in the fish as the likely cause of death. To minimize suffering and distress, the fish that survived to day 21 post-infection were euthanized using a lethal dose of Tricaine methanesulfonate, MS 222 (Sigma), concentration of 200 mg L^-1^, for at least 5 minutes prior to sampling of fin clips. The survivors had to be sacrificed because they are carriers of the pathogen and hence pose a bio-security risk to other naïve fish cohorts. Fin clips from all mortalities and survivors were individually kept in 95% ethanol until DNA was extracted using established protocols [23].

### BCWD resistance phenotypes

Fish survival was evaluated for 21 d post-challenge with survivors being assigned a value of 22 d post-challenge. Each individual fish had a record of survival days, the number of days to death post-challenge with *Fp*. Each individual fish also had a record of survival status. The binary survival status phenotype had two classes: 2 = fish was alive on day 22 post-challenge and 1 = fish died during the 21 d post challenge evaluation period. In the QTL analysis, we expected that survival days and status phenotypes would enable mapping QTL affecting resistance or susceptibility to BCWD.

### QTL mapping families

Two F_2_/BC_1_ outbred line-cross QTL mapping families (2009070 and 2009196) that we previously analyzed using genome scans with microsatellites [4] were genotyped in this study. From the 252 and 200 offspring that we challenged from family 2009070 and 2009196, respectively, we selected 60 first to die and randomly selected 60 survivors for genotyping with RAD SNPs (a total of 120 offspring per family).

### RAD genotyping

Genomic DNA from grandparents, parents and offspring of mapping families 2009070 and 2009196 was digested with restriction enzyme *SbfI*, and RAD sequencing libraries were prepared as previously described [11]. Each RAD library containing 30 to 32 indexed samples with a unique six-nucleotide barcode for each sample was sequenced (single end 100bp read) on a single lane of HiSeq 2000. Raw sequences were submitted to the short read archive of GenBank under accession number SRP055465 (SRA: SRS856160-SRS856447). The sequence reads were processed to identify SNPs using Novoalign and Perl scripts as previously described [5]. For sequence alignment we trimmed the first 12 bp, including the indexed barcodes and the restriction enzyme recognition site. In addition we trimmed the next two bp (positions 13–14) and the last 5 bp (positions 96–100) due to lower sequence quality scores across reads and individual samples, which can increase the false SNPs discovery rate [11]. To ensure sufficient sequence coverage in the parents, each parent was sequenced 3–5 times to generate 8-12M sequence reads per parent for the RAD analysis. The parental genotypes were called using the bioinformatics pipeline of SNP discovery and genotype scoring as previously described [11]. For the offspring and grandparents genotype scoring, the RAD sequences were mapped to the parental alleles of each SNP requiring exact matching in Novoalign. For each offspring we required a minimum of four identical sequence reads to call it homozygous for that SNP. For a heterozygous genotype call we required that the total number of reads for the locus (e.g. both alleles) will be at least four, and that the frequency of the minor allele sequence reads (MAF) will be from 10% to 50%. If both alleles were present in the offspring sample, and the MAF was smaller than 10%, we did not call a genotype for that particular SNP in that particular offspring and it was classified as missing data. Chi-square goodness-of-fit tests were used to check the Mendelian segregation ratio of each SNP, and SNPs with significant Bonferroni-corrected segregation distortions (P < 1e-5) were dropped from the final genotype dataset.

### Linkage analyses

The RAD SNP genotype data were combined with the microsatellite genotype data from our previous report [4]. Markers were mapped onto 28 autosomes and one sex chromosome using MULTIMAP version 2.0 [24]. The recombination fractions were converted to genetic distances by means of Kosambi’s mapping function with the assumption of incomplete interference allowing some double crossovers [25]. Based on two-point analyses, the markers were assigned to linkage groups using LOD ≥ 10 and a maximum recombination fraction of 0.2. Using microsatellites as anchors, linkage groups were named according to the reference genetic maps of rainbow trout [26,27]. For the remaining unlinked markers, the MULTIMAP/CRIMAP best-twopoints function was used to find out if some of them can be assigned to a linkage group at LOD ≥ 9. The initial linkage map for each linkage group was constructed using LOD ≥ 5, then additional markers were added using LOD ≥ 4 and then again using LOD ≥ 3.

### Genome wide association analyses

For the association analyses, SNP loci and offspring with more than 30% missing data (call rate <70%) were removed from the dataset, reducing the sample size in family 2009070 to N = 118. Genome wide association analysis for each family was performed using the Grammar-Gamma method [28] implemented in the R package GenABEL [29]. This method runs a single-point association test accounting for family relatedness and includes two analysis steps. First, we performed polygenic analysis modeling family relationships; the genomic kinship matrix estimated from SNPs was included in the model of association analysis. Second, we performed association analysis using the residuals from step 1 analysis, so pure SNPs effect were used in the association tests. For simplicity, we used a Bonferroni correction and hence only markers with *P* <0.00001 for the phenotypes of survival days or survival status were defined as significant SNPs associated with BCWD.

### QTL analysis

The QTL genome scans were performed using the outbred population TREE analysis module from the software GridQTL [30] following the analysis steps and model we have previously described [4]. The TREE analysis module can be used to analyze a three-generation pedigree consisting of a single large F_2_ or BC_1_ family (with a maximum of four grandparents and two F_1_ parents). With this population structure, the genetic model does not need to assume fixation of the QTL alleles in the grandparental generation, because up to four different alleles at any one location can be modeled [31].

The genome-wide significance threshold level of detected QTL was estimated with this expression: *P*_*GenomeWide*_ = 1−(1−P)^1/*r*^ [32]; here *P* is the nominal *P*-value estimated assuming an approximate *F* distribution with numerator and denominator DF as number of parents and progeny, respectively; and *r* is the proportion of total genome length attributed to the chromosome. The detected QTL was defined as significant if *P*_*GenomeWide*_<0.05. The proportion of phenotypic variance explained by each QTL $(h_{q}^{2})\;$ was calculated as 2[1−(*MSE*_*full*_/*MSE*_*reduced*_)] where *MSE*_*full*_ is the mean squared error of the full model, accommodating one QTL effect for each informative mapping parent, while *MSE*_*reduced*_ is the mean squared error of the reduced model omitting the QTL effect [33].

### Search for positional candidate genes

To obtain the genome sequences of scaffolds near the significant BCWD QTL regions, the flanking sequences of SNP and microsatellite markers associated with BCWD QTL were used as queries of BLASTN searches on the recently published reference genome [22], requiring exact match for the SNP sequences except the SNP site, or more than 95% sequence identity with the sequences of microsatellite markers. The QTL positive scaffolds were then examined for neighboring putative candidate genes based on whether they were previously identified as differentially expressed genes or expressed immune-related genes identified in our whole-body RNA-seq study [7]. We maintained the annotation and GO terminology from the Marancik et al. 2015 study.

## Results and Discussion

### RAD SNP genotypes and linkage maps

In mapping family 2009070 we scored 5,612 RAD SNPs, which were combined with 267 microsatellites from our previous study [4] for linkage analysis. Using two-point analysis a total of 5,851 markers were assigned to the rainbow trout 29 chromosomes based on our previous microsatellites assignments. Of those, 5,221 SNPs with call rate (CR) > 0.70 were used for genome-wide association study (GWAS) and a total of 1,167 markers were ordered on chromosomes using a LOD score ≥ 3 threshold to generate a genetic map for QTL mapping. The total sex-averaged map length was 3,588 cM with an average marker spacing of 3.24 cM (S1 File).

In mapping family 2009196 we scored 4,946 RAD SNPs, which were combined with 271 microsatellites from our previous study [4] for linkage analysis. Using two-point analysis a total of 5,202 markers were assigned to the rainbow trout 29 chromosomes. Of those, 4,623 SNPs with CR > 0.70 were used for GWAS and a total 1,384 markers were ordered on chromosomes using a LOD score > 3 threshold to generate a genetic map for QTL mapping. The total sex-averaged map length was 3,961 cM with an average marker spacing of 3.05 cM (S2 File).

### Genome wide association analyses

**Fig 1:**
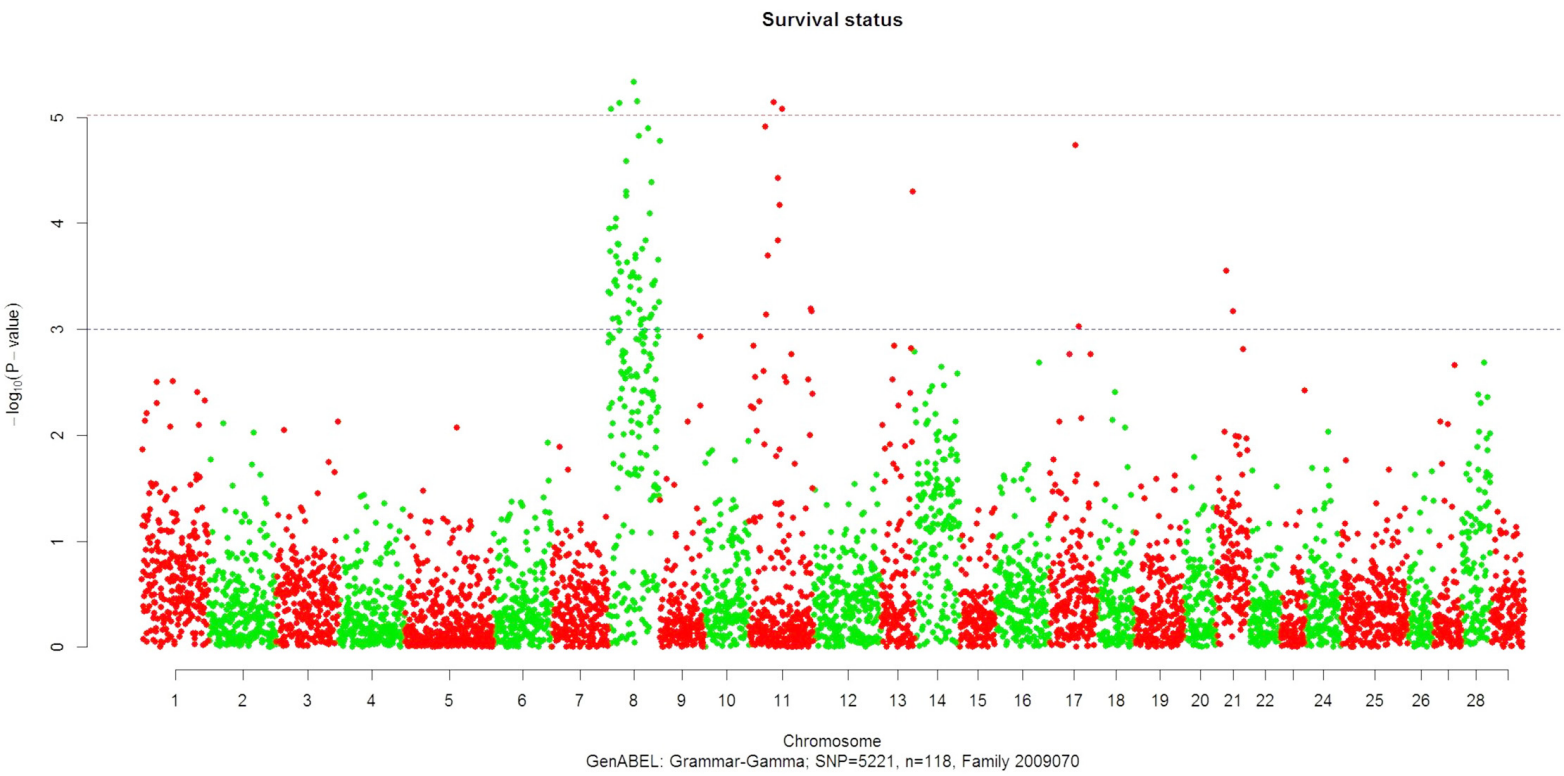
Results of GWAS for the survival status phenotype recorded post-infection with *Flavobacterium psychrophilum* in rainbow trout family 2009070.

**Fig 2:**
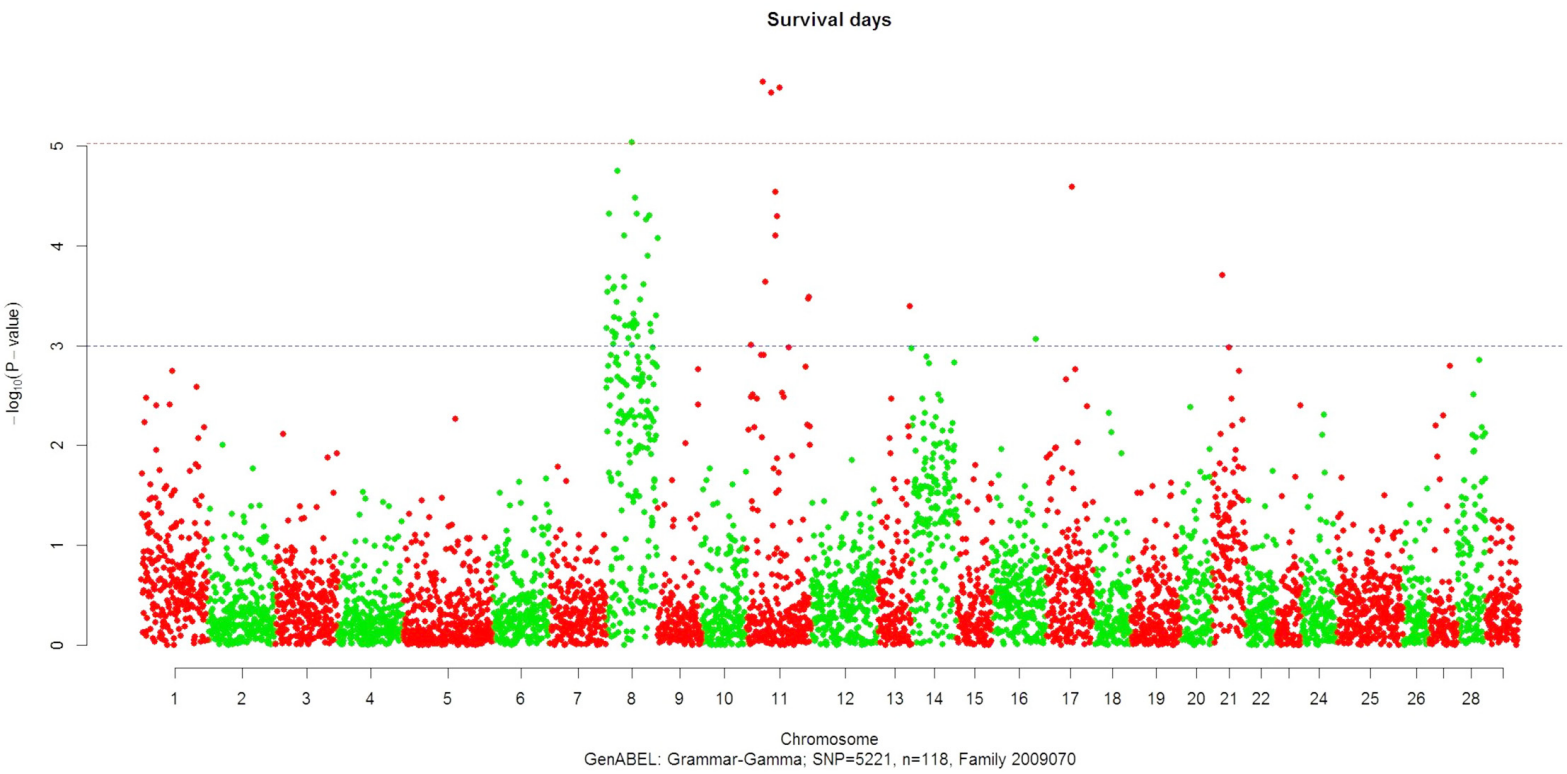
Results of GWAS for the survival days phenotype recorded post-infection with *Flavobacterium psychrophilum* in rainbow trout family 2009070.

Because of the increased number of markers in this study, we used GWAS to identify individual markers with the strongest effect on BCWD resistance in the two genotyped families. Seven SNPs were found to have a significant effect on BCWD resistance in family 2009070 using the stringent Bonferroni correction threshold of *P*_*DF1*_ < 0.05/5,221 (9.6E-06) or approximately *P* <0.00001 (Table 1 and Figs 1 and 2). Four of the significant SNPs were located on chromosome Omy8, confirming our previous finding of a significant QTL on this chromosome in that family [4]. The other three SNPs were mapped to Omy11. This is somewhat surprising as previously we did not map a QTL on Omy11 in this family. However, a suggestive QTL was previously mapped to this chromosome using microsatellites in the related family 2009044, and a significant QTL was indeed mapped to this chromosome in this study (Table 2A and see text below). It is likely that the addition of the large number of RAD SNPs in this study improved the marker coverage on or near this major QTL on chromosome 11 in family 2009070, and hence enabled the detection of this previously undetected QTL. No significant RAD SNP association with BCWD resistance was detected in family 2009196.

**Table 1:** GWAS summary: SNPs associated with BCWD resistance in rainbow trout family 2009070^a^.

				Survival Status		Survival Days	
Omy	SNP	Allele 1	Allele 2	Effect^b^	*P*_*DF1*_ ^c^	Effect^b^	*P*_*DF1*_ ^c^
8	RM20090703355	A	G	0.58	4.6E-06*	8.5	9.4E-06*
8	RM20090703790	T	C	0.53	7.1E-06*	7.4	3.4E-05
8	RM20090701338	T	C	-0.57	7.3E-06*	-8.3	1.8E-05
8	RM20090700278	G	A	-0.41	8.4E-06*	-5.7	4.9E-05
11	RM20090703064	C	G	-0.54	7.2E-06*	-8.5	3.0E-06*
11	RM20090703983	G	T	-0.56	8.4E-06*	-9.0	2.6E-06*
11	RM20090702086	T	G	-0.36	1.2E-05	-5.9	2.3E-06*

^a^ N = 118 offspring genotyped with 5,221 RAD SNPs (CR>0.70).

^b^ Effect of allele 2 on the corresponding trait phenotype.

^c^ Association of SNP with a disease survival phenotype is significant if *P*_*DF1*_ < 0.05/5,221 (9.6E-06).

An asterisk (*) marks the significant association values we detected.

**Table 2:** QTL for BCWD resistance detected and validated using outbred population (TREE Model) analysis in two rainbow trout families for the phenotypes survival days (Days) and survival status (Status).

Omy	Phenotype	Position (cM)	*F* value ^b^	*P*_*GenomeWide*_ ^b^	Effect (h^2^_q_)	95% CI (cM)	Left flanking marker	Right flanking marker	Previous ^c^
**A. Family 2009070** ^a^ **:**									
1	Days	83	6.9	4.3E-02	0.09	24–86	RM20090706108	RM20090701492	Suggestive
7	Status	54	12.9	1.3E-04	0.18	17–143	RM20090702856	RM20090700328	Suggestive
7	Days	142	8.4	7.8E-03	0.11	28–149	RM20090700643	RM20090701238	Suggestive
8	Days	47	32.1	3.3E-11	0.44	7–88	RM20090700197	OMM1295	Significant
8	Status	47	43.7	8.9E-15	0.58	1–88	RM20090700197	OMM1295	Significant
8	Days	88	15.1	3.2E-05	0.21	0–88	RM20090701162	RM20090700382	Suggestive
8	Status	88	21.0	2.1E-07	0.30	9–88	RM20090701162	RM20090700382	Suggestive
11	Days	122	14.3	4.0E-05	0.20	24–137	RM20090702086	RM20090700457	None
11	Status	122	10.8	9.1E-04	0.15	11–137	RM20090702086	RM20090700457	None
14	Days	113	8.0	1.4E-02	0.11	2–114	RM20090701114	OMM5174	None
25	Status	57	8.2	1.1E-02	0.11	9–105	OMM1532	RM20090706025	None
25	Days	59	11.8	4.1E-04	0.17	4–103	OMM1532	RM20090706025	Suggestive
**B. Family 2009196** ^a^ **:**									
6	Days	23	7.73	1.5E-02	0.18	12–115	RM20091960212	RM20091961885	Significant
6	Status	23	7.93	1.2E-02	0.19	8–82	RM20091960212	RM20091961885	Significant
8	Status	9	9.76	4.9E-02	0.17	0–109	RM20091961322	RM20091960210	None
12	Days	108	8.71	6.4E-03	0.21	14–120	OMM5152	RM20091962540	Significant
12	Status	109	7.70	1.6E-02	0.18	17–117	OMM5152	RM20091962540	Significant

^a^ N = 120 offspring genotyped in each family with 1,167 and 1,348 mapped markers (microsatellites and RAD SNPs) in families 2009070 and 2009196, respectively.

^b^ Only QTL found to be significant in the current study are listed (*P*_*GenomeWide*_<0.05).

^c^ QTL was previously detected as significant, suggestive or not detected in this family using microsatellites only [4].

In this study we applied GWAS using individual families as the target population to maximize the number of informative RAD SNPs for the analysis. However, the relatively small sample size (N = 120 per family) hindered our ability to find SNPs with small to moderate effect on the trait. Typically, GWAS studies are conducted using large breeding populations of multiple families with higher marker density to ensure good representation of the entire genome [34]. Recently, a rainbow trout 57K SNP chip was developed [35], which will lead to more opportunities to conduct GWAS in rainbow trout. However, the use of RAD sequencing remains a viable option as the cost of SNP chip genotyping for a large number of fish remains a major constraint for many labs, and for some of the other farmed fish species a SNP chip is not available yet.

### QTL mapping

As the main purpose of this study was to validate the QTL we have previously mapped using microsatellites in mapping families 2009070 and 2009196, we only present the results for significant QTL (*P*_*GenomeWide*_<0.05) in Table 2. Eight and three significant QTL were detected in families 2009070 and 2009196, respectively. As expected, the major QTL in family 2009070 was located at position 47 cM on chromosome Omy8 explaining 58% and 44% of the phenotypic variance for survival status and days, respectively. However, it is important to note here that due to selective genotyping our estimates of the QTL effects in the current study are likely up-biased [36]. This QTL was the only significant QTL we detected in our previous genome scan in this family [4]. The other Omy8 QTL we detected at position 88 cM was not detected in family 2009070 in the previous genome scan, but two distinct suggestive QTL were previously detected on Omy8 in a related family. The two distinct suggestive QTL we have previously detected on Omy7 in this family were validated in the current genome scan. Similarly, the previously detected suggestive QTL on Omy1 and 25 were confirmed here, although the Omy25 QTL was only detected for the survival days’ phenotype in the previous study. The Omy22 QTL which was also detected in the previous genome scan in family 2009070 was the only suggestive QTL that was not confirmed in this study. In addition, we have also detected two new QTL for this family on Omy11, which is consistent with the GWAS results, and on Omy14. The likely reason for detecting the two novel QTL on Omy11 and 14 in the current study is the increased marker density compared to the previous microsatellites-based genome scans.

In family 2009196, the two previously detected significant QTL on Omy6 and 12 were confirmed and a new QTL was detected on Omy8. However, the previous suggestive QTL in this family on chromosomes Omy5, 13, 19, 20 and 24 were not confirmed in the current study. The dichotomy between the results we obtained from families 2009070 and 2009196 using the improved marker density is puzzling. In the first, five of six suggestive QTL were validated by the improved density, while in the latter, all the five suggestive QTL were absent. One possible explanation is the stronger impact of the selective genotyping upwards bias on estimating QTL effects in family 2009070. In this family, the offspring sample size was reduced from 252 in the previous genome scan to 120, while in family 2009196 it was only reduced by 80 samples (from 200 to 120 offspring). Another possible explanation is that the very large effect of the major Omy8 QTL in family 2009070 masked the impact of the other QTL in that family in the microsatellites genome scan, but the improved markers density in the current study enabled greater detection power of the smaller effect, yet significant, QTL. Whatever the reason for the discrepancy between families with respect to validation of the suggestive QTL; the main conclusion is that all the significant QTL from our microsatellites genome scans were validated after increasing the marker density and genome coverage.

Previously our group reported QTL for handling and confinement stress response using microsatellites and RAD SNPs genome scans in an F_2_ mapping family [5,6]. The major stress QTL were mapped to chromosomes Omy8 and 12. Similarly to the current BCWD resistance study, in the stress study we detected two distinct peaks on the Omy8 QTL plots. The mapping family used in the stress studies was from the even-year class of the NCCCWA breeding program and hence unrelated to the families used here for BCWD QTL mapping. To simplify the terminology we will define the family 2009070 Omy8 QTL located at 47 cM in the current study as QTL-1 and the one located at 88 cM as QTL-2. Comparison of the microsatellites flanking the two QTL revealed that both are co-localized with the stress QTL. QTL-1 for both traits is near microsatellite OMM1295 and QTL-2 is at the chromosome end beyond microsatellite marker BX870052a (S1 File). Direct comparison between RAD SNPs is harder as direct sequence alignment of the SNPs that flank the QTL for the two traits revealed that they are not the same markers. However through matching of the RAD SNP sequences with the SNP chip sequences [35] we were able to place the two Omy8 QTL for BCWD and for stress on the new high density SNPs genetic map that is based on genotypes from the 57K SNP chip (Baranski et al., in preparation). Using this consensus map, we confirmed that RAD SNPs that flank the stress and BCWD QTL-1 are completely linked on the male map and are within 2.5 cM on the female map, while the RAD SNPs neighboring QTL-2 for both traits were mapped to the terminal end of the chromosome on the female map (data not shown). Recently, Danzmann et al. (in preparation) has reported the mapping of a QTL for growth that is co-localized to the same SNPs that flank Omy8 QTL-1 for stress and BCWD in yet another unrelated rainbow trout population. Stress can have a negative impact on fish performance including decreased growth rates, compromised feed conversion efficiencies, increased disease incidence and mortality [37,38]. Hence it is possible that a single QTL can have pleiotropic effects on stress, growth and disease resistance. However, at this time we cannot exclude the possibility that the QTL for each trait are different and that they merely co-localize in physical proximity on the same chromosome as we have found to be the case with the Omy19 QTL for BCWD and spleen size in another mapping population at the NCCCWA [39,40].

The Omy12 BCWD QTL in family 2009196 is also co-localized with the previously identified stress response QTL on that chromosome [5]. The BCWD is flanked by microsatellite OMM5152 and RAD SNP RM20091962540 (Table 2B). OMM5152 is also tightly linked to the stress QTL and RM20091962540 can be mapped to scaffold_7717 of the rainbow trout reference genome [22], which is the same scaffold that harbors one of the stress response-associated RAD SNPs (RD20080521532). However, the RAD sequence flanking the BCWD SNP is different from the sequence of the stress RAD SNP. Here again, it is possible that the same QTL has a pleiotropic effect on the two traits, but it may also be a case of mere physical linkage between two different QTL.

In a separate study, Campbell et al. used RAD SNPs for genome-wide association analyses with BCWD resistance in a commercial rainbow trout breeding population [19]. To compare their results to the current study we aligned the RAD sequences of all the SNPs that they found to be associated with BCWD with the RAD SNP sequences from the current study. We have found seven matches with their RAD SNP sequences, but only one of them, R14353, matched the sequence of one of our markers that was mapped near the Omy6 QTL in family 2009196. That RAD SNP, RM20091964665, is located 34 cM on the sex-averaged map from the nearest QTL flanking marker on our Omy6 genetic map, but only 16 cM away on the female map for that chromosome (marker RM20091961885; S2 File, Sheet Omy6). With the currently available information we cannot determine if the same Omy6 QTL was detected by both studies. Repeating the association study on the same commercial population with the new 57K SNP chip may resolve this question.

### Positional candidate genes

BLAST sequence identity searches using the QTL and GWAS markers from families 2009070 and 2009196 detected 23 genome scaffold hits (Table 3). The 23 scaffolds cover a combined size of approximately 16.5 Mb, which is ~0.09% of the current reference genome physical size. Of the 23 scaffolds, 18 contained predicted open reading frames (ORFs) with a combined total of 418 ORFs, which is ~0.09% of the current reference genome predicted ORFs [22]. S3 File includes all the ORFs found in the GWAS- and QTL-positive genome scaffolds and their putative sequence similarity description and GO annotation [7]. Only one of the genome scaffolds that matched QTL markers was large enough to harbor the two QTL peak-flanking markers. It is scaffold_4 (~4 Mb; 108 ORFs) which matches the two Omy8 QTL-1 flanking markers in family 2009070.

**Table 3:** Rainbow trout genome scaffolds [22] matched by markers linked to BCWD resistance QTL.

Omy^a^	Family	Marker	Scaffold	Scaffold Position	Scaffold Size	ORFs in Scaffold
1	2009070	RM20090701492	scaffold_36	1,432,000	2,327,712	57
6	2009196	RM20091960212	scaffold_607	39,872	631,915	11
6	2009196	RM20091961885	scaffold_7428	12,482	31,380	N/A
7 QTL-1	2009070	RM20090702856	scaffold_446	730,023	808,737	27
7 QTL-1	2009070	RM20090700328	scaffold_318	240,939	973,806	28
7 QTL-2	2009070	RM20090700643	scaffold_6249	8,845	37,609	N/A
8 GWAS	2009070	RM20090703355	scaffold_886	255,797	441,629	8
8 GWAS	2009070	RM20090703790	scaffold_262	763,259	1,102,120	26
8 GWAS	2009070	RM20090701338	scaffold_172	321,650	1,393,283	38
8 GWAS	2009070	RM20090700278	scaffold_45489	2,239	3,014	1
8 QTL-1	2009070	RM20090700197	scaffold_4	3,238,320	4,008,788	108
8 QTL-1	2009070	OMM1295	scaffold_4	3,898,403	4,008,788	108
8 QTL-2	2009070	RM20090701162	scaffold_1339	230,929	258,856	8
8 QTL-2	2009070	RM20090700382	scaffold_246	264,303	1,146,030	34
8	2009196	RM20091961322	scaffold_4980	36,492	46,256	N/A
11 GWAS	2009070	RM20090703064	scaffold_1831	11,242	165,043	3
11 GWAS	2009070	RM20090703983	scaffold_1381	2,31	248,913	9
11 QTL	2009070	RM20090700457	scaffold_237	1,138,754	1,173,938	18
12	2009196	OMM5152	scaffold_9847	18,348	22,839	N/A
12	2009196	OMM5152	scaffold_79937	415,938	466,645	14
12	2009196	RM20091962540	scaffold_7717	3,874	30,067	2
14	2009070	RM20090701114	scaffold_469	307,434	778,904	13
25	2009070	OMM1532	scaffold_5253	20,223	44,156	N/A
25	2009070	RM20090706025	scaffold_913	249,859	426,771	13

^a^ In family 2009070 on Omy7 and 8 we have identified two QTL. Omy7 QTL-1 and -2 are located on cM 54 and 142, respectively. Omy8 QTL-1 and -2 are located on cM 47 and 88, respectively. GWAS notation near the Omy number means that the marker was detected through genome-wide association analysis.

From the 418 predicted ORFs, we applied further filtering to identify potential candidate genes. Previously, we have conducted whole-body RNA-seq and gene expression experiments on the response to *Fp* injection in genetically divergent lines of rainbow trout (Marancik et al., 2015). S1 Table lists a combined total of 100 putative candidate genes from the Marancik et al. study that mapped to 16 of the QTL-positive genome scaffolds in this study. These 100 putative positional candidate ORFs include genes that based on their predicted functions are likely to be involved in immune response or the response to chronic stress. One of them is the suppressor of cytokine signaling (SOCS) 6 ORF (reference sequence ID GSONMT00073706001), which is located on scaffold_246 near the co-localized Omy8 QTL-2 for stress-response and BCWD-resistance. The SOCS genes encode proteins that negatively regulate the JAK/STAT signaling pathway, and as a consequence are involved in negative regulation of growth and cytokine-mediated immune response. Recently Philip and Vijayan [41] observed upregulation of SOCS-1 and -2 transcripts levels in response to cortisol in the rainbow trout liver; and suggested that the associated reduction in the JAK/STAT pathway may lead to growth reduction and immune suppression during stress in trout. However, SOCS-6 mRNA expression in rainbow trout is not regulated by immune stimuli that upregulate SOCS-1 and -2 mRNA expression and the role of its functional divergence is currently unknown [42]. Another ORF which may be involved in the JAK/STAT pathway is tyrosine-protein kinase jak1 (GSONMT00077835001), which is located on scaffold_4, but just outside the sequence segment that is flanked by the two Omy8 QTL-1 markers (Table 3). Two ORFs with possible involvement in immune response or signal transduction that are located within the segment between scaffold_4 positions 3,238,320 and 3,898,403 are b-cell lymphoma 6 protein homolog (GSONMT00077908001) and receptor-interacting serine threonine-protein kinase 2 (GSONMT00077890001).

Other putative genes with potential function related to the immune response to infection or signal transduction that are located near QTL that we have mapped in this study on Omy1 scaffold_36 are transcription factor jun-b-like (GSONMT00077443001), nuclear factor interleukin-3-regulated (GSONMT00077479001) and interleukin enhancer-binding factor 3 homolog isoform x1 and x3 (GSONMT00077466001 and GSONMT00077465001). On Omy6 scaffold_607 toll-like receptor 20 or TLR 13-like (GSONMT00038184001). On Omy11 scaffold_1381 complement component c8 gamma chain precursor (GSONMT00057235001) and on scaffold_237 neutrophil cytosol factor 2-like (GSONMT00073143001). On Omy14 scaffold_469 tyrosine-protein kinase btk (GSONMT00017181001). On Omy25 connector enhancer of kinase suppressor of ras 3 (GSONMT00031747001).

Previously we have identified 316 liver differentially expressed transcripts (DETs) in response to handling and confinement stress [43]. Liu et al. (2015) has also mapped three of those stress-response DETs homologous to a serine/threonine protein kinase SBK1 (NP_998006.1 in zebrafish) to chromosome Omy12 near microsatellite OMM5152, which borders the co-localized stress-response and BCWD-resistance QTL on Omy12. Hence, this SBK1-like ORF is another putative positional candidate gene with potential pleiotropic effects on stress response and disease resistance in rainbow trout. Another one of those liver stress-response DETs, g05592.1 which is 99% identical to the reference ORF GSONMT00077855001 (regulator of g-protein signaling 13-like), is located on scaffold_4 near the co-localized Omy8 QTL-1 peak-flanking markers from the current study.

## Conclusions

The use of RAD SNPs in the genome scans increased the number of mapped markers from ~300 to ~5,000 per family. All previously detected significant QTL were validated using the new RAD markers genotyping system. The increased marker density enabled detection of new QTL and validation or rejection of previously detected suggestive QTL, although we cannot rule out that the selective genotyping we used in the current study might have up-biased the effects of the detected QTL. The significant QTL detected in the previous genome scan on chromosome Omy8 in family 2009070 was validated, and explained up to 58% of the phenotypic variance in this family. In addition, a second QTL was detected on Omy8 in this family. Two novel QTL on Omy11 and 14 were also detected in family 2009070 and the previously suggestive QTL on Omy1, 7 and 25 were validated. In family 2009196, the significant QTL on Omy6 and 12 were validated and a new QTL on Omy8 was detected, but none of the previously detected suggestive QTL were validated. The two Omy8 QTL from family 2009070 and the Omy12 QTL from family 2009196 were found to be co-localized with handling stress response QTL that our group has previously identified, but from the currently available data we cannot determine if the co-localized QTL are the result of genes with pleiotropic effects or mere physical proximity. The genetic markers linked to BCWD resistance QTL were used to query the scaffolds of the rainbow trout reference genome assembly and the QTL-positive scaffold sequences were found to contain 100 putative candidate genes from our previous RNA-seq study. Several of the candidate genes located on or near the two Omy8 QTL detected in family 2009070 may be involved in stress response with potential negative regulation of immune response and growth in rainbow trout. The candidate genes we identified in this study merit further evaluation, and in addition, we do not rule out the potential contribution of encoded miRNA or lncRNA within the QTL regions. However, prior to conducting expensive functional studies it is important to assess the wider impact of the QTL in other rainbow trout families and breeding populations. We are currently conducting genome-wide association studies using the new 57K SNP chip with larger sample size in multiple families from the NCCCWA breeding population and from a commercial breeding population. The results of those additional studies will allow for evaluation of the impact of the detected QTL in other aquaculture populations and possibly more precise fine-mapping the BCWD resistance QTL.

## Supporting Information

S1 File Family 2009070 marker chromosome assignments and genetic maps. (XLSX)

S2 File Family 2009196 marker chromosome assignments and genetic maps. (XLSX)

S3 File Open reading frames mapped to QTL sequence scaffolds. (XLSX)

S1 Table Candidate genes from RNA-seq experiments and gene expression studies (Marancik et al., 2015) that are mapped near BCWD QTL. (XLSX)
